# Air Pollution Exposure Is Associated With Cognitive Performance: Results From the Einstein Aging Study

**DOI:** 10.1093/geroni/igab046.531

**Published:** 2021-12-17

**Authors:** Nelson Roque, Charles Hall, Mindy Katz, Martin Sliwinski

**Affiliations:** 1 University of Central Florida, Orlando, Florida, United States; 2 Albert Einstein College of Medicine, Bronx, New York, United States; 3 The Pennsylvania State University, University Park, Pennsylvania, United States

## Abstract

Prior research has established that those exposed to higher levels of fine particulate matter (PM1, PM2.5) air pollution have higher levels of accumulated amyloid-beta (Aβ) and tau in frontal cortex at autopsy, higher error rates on cognitive function assessments, and lower scores on memory and both verbal and non-verbal intelligence assessments. We explored the relationship between regional air quality monitoring measures (EPA AirData) and baseline cognitive performance of 312 older adults, from the Einstein Aging Study (EAS, NIA P01AG003949). Participants completed neuropsychological assessments at baseline and each followup wave (i.e., delayed free recall and total recall; Trails A & B, Digit Symbol substitution task (DSST), MoCA). For each participant, based on their zipcode, we computed average PM2.5 exposure at various exposure windows (1-15, 30-60, 60-90, 90-120 days prior to baseline). Adjusting for age, education, and gender across all models, mean of daily particulate matter exposure at various exposure windows (30-60, 60-90 days) was significantly related to performance on the MoCA and Trails A & B, in expected directions (i.e., higher pollution, worse cognitive performance - more error, slower speed). Models with memory performance as the outcome indicated that only distant time horizons were related to memory performance (i.e., 60-90, 90-120 days prior). These findings suggest that particulate matter air pollution likely affects different cognitive domains at different timescales. This methodology cannot address contributions from indoor air quality and mobility - an exposure misclassification likely resulting in significant biases towards the null in the estimation of the effects of air pollution.

